# Quantifying the Extent to Which Successful Juniors and Successful Seniors are Two Disparate Populations: A Systematic Review and Synthesis of Findings

**DOI:** 10.1007/s40279-023-01840-1

**Published:** 2023-04-06

**Authors:** Arne Güllich, Michael Barth, Brooke N. Macnamara, David Z. Hambrick

**Affiliations:** 1 Department of Sports Science, University of Technology Kaiserslautern, Erwin-Schrödinger-Straße 57, 67663 Kaiserslautern, Germany; 2 Department of Sport Science, Universität Innsbruck, Fuerstenweg 185, 6020 Innsbruck, Austria; 3 Department of Business and Society, University of Applied Sciences Kufstein Tyrol, Andreas Hofer-Straße 7, 6330 Kufstein, Austria; 4 Department of Psychological Sciences, Case Western Reserve University, 11220 Bellflower Road, Cleveland, OH 44106 USA; 5 Department of Psychology, Michigan State University, 316 Physics Road, East Lansing, MI 48825 USA

## Abstract

**Background:**

To what extent does the pathway to senior elite success build on junior elite success? Evidence from longitudinal studies investigating athletes’ junior-to-senior performance development is mixed; prospective studies have reported percentages of juniors who achieved an equivalent competition level at senior age (e.g., international championships at both times) ranging from 0 to 68%. Likewise, retrospective studies have reported percentages of senior athletes who had achieved an equivalent competition level at junior age ranging from 2 to 100%. However, samples have been heterogeneous in terms of junior age categories, competition levels, sex, sports, and sample sizes.

**Objective:**

This study aimed to establish more robust and generalizable findings via a systematic review and synthesis of findings. We considered three competition levels—competing at a national championship level, competing at an international championship level, and winning international medals—and addressed three questions: (1) How many junior athletes reach an equivalent competition level when they are senior athletes? (2) How many senior athletes reached an equivalent competition level when they were junior athletes? The answers to these questions provide an answer to Question (3): To what extent are successful juniors and successful seniors one identical population or two disparate populations?

**Methods:**

We conducted a systematic literature search in SPORTDiscus, ERIC, ProQuest, PsychInfo, PubMed, Scopus, WorldCat, and Google Scholar until 15 March 2022. Percentages of juniors who achieved an equivalent competition level at senior age (prospective studies) and of senior athletes who had achieved an equivalent competition level at junior age (retrospective studies) were aggregated across studies to establish these percentages for all athletes, separately for prospective and retrospective studies, junior age categories, and competition levels. Quality of evidence was evaluated using the Mixed Methods Appraisal Tool (MMAT) version for descriptive quantitative studies.

**Results:**

Prospective studies included 110 samples with 38,383 junior athletes. Retrospective studies included 79 samples with 22,961 senior athletes. The following findings emerged: (1) Few elite juniors later achieved an equivalent competition level at senior age, and few elite seniors had previously achieved an equivalent competition level at junior age. For example, 89.2% of international-level U17/18 juniors failed to reach international level as seniors and 82.0% of international-level seniors had not reached international level as U17/18 juniors. (2) Successful juniors and successful seniors are largely two disparate populations. For example, international-level U17/18 juniors and international-level seniors were 7.2% identical and 92.8% disparate. (3) Percentages of athletes achieving equivalent junior and senior competition levels were the smallest among the highest competition levels and the youngest junior age categories. (4) The quality of evidence was generally high.

**Discussion:**

The findings question the tenets of traditional theories of giftedness and expertise as well as current practices of talent selection and talent promotion.

A PRISMA-P protocol was registered at https://osf.io/gck4a/.

**Supplementary Information:**

The online version contains supplementary material available at 10.1007/s40279-023-01840-1.

## Key Points

**Table:** 

Successful junior athletes and successful senior athletes are largely two disparate populations.
Most successful junior athletes do not achieve an equivalent competition level when they are senior athletes (e.g., international competition level as a junior and a senior).
Most successful senior athletes did not achieve an equivalent competition level when they were junior athletes (e.g., international competition level as a junior and a senior).

## Introduction

To what extent does the pathway to senior elite success build on junior elite success? This question is a subject of debate in sports science, medicine, and psychology. A range of views have been advanced in the scientific literature, with two views at opposite extremes. One view emphasizes the importance of a high level of youth performance (e.g., national level and above) for later achievement of a high level of senior performance, while the other view suggests limited importance of a high level of youth performance for later senior high performance.

The first view assumes that a high level of performance in the early years of one’s career is an important precondition for the long-term development of adult elite performance and implies that successful juniors and successful seniors are largely one identical population. This is a critical premise of both giftedness and expertise theories (e.g. [1–4]). For example, giftedness has been operationally defined as outperforming 90% or more of one’s peers at a young age (e.g. [2, 4]). Relatedly, according to the deliberate practice view of expertise, “a high level of performance […] will always be the best predictor of future performance” [1, p. 393]. Furthermore, according to this view, “the best training environments with master teachers and coaches carefully select the individuals with the best performance in late adolescence” [1, p. 393].

Likewise, several applied researchers and practitioners in sports have postulated that junior elite performance is critically important in an athlete’s pathway towards senior elite performance (i.e., in the highest, open-age category [5–12]). For example, Hollings and Hume [8] claimed that “an athlete has to be very good as a junior in order to be very good as a senior athlete” [p. 132]. Hollings and Hume [8] recommended that to produce successful senior athletes, sport systems should concentrate their resources on junior athletes who have reached finals and medals at junior world championships.

This view corresponds with international sport policies and practices. In the 1970s–1980s, major sports began introducing continental and world junior championships in the oldest junior age category in each sport, ages 16–17, 17–18, or 18–19 years, respectively [13]. Today, the websites of international sport federations show that many sports have established continental and world championships, festivals, and circuits at ages as young as 11–15 years. Relatedly, national sport systems funnel resources into talent promotion programs, which typically select the most advanced young athletes and, once selected, seek to further accelerate their adolescent performance development (for reviews, see [14, 15]).

In contrast to this view, the second view holds that junior performance has limited importance for the development of later, senior performance and implies that successful juniors and successful seniors are largely two disparate populations. This view is based on four lines of argument.

1. Predictors of junior performance are not necessarily the same as—and indeed are partly opposite of—predictors of senior performance. This has been demonstrated in recent meta-analyses [16, 17]. In particular, compared with lower-performing junior athletes, higher-performing juniors started playing their main sport at a younger age, engaged in greater amounts of coach-led specialized practice in their main sport, and engaged in less other-sports practice [16, 17]. By contrast, the opposite pattern predicted the greatest senior elite athletes. Compared with national-class senior athletes, world-class senior athletes started playing their main sport at a later age, engaged in less coach-led practice in their main sport during childhood and adolescence, and engaged in greater amounts of other-sports practice during childhood and adolescence [16, 17]. Relatedly, higher-performing junior athletes reached developmental performance-related ‘milestones’ (first national championships, first international championships) at a younger age than lower-performing juniors, whereas senior world-class athletes had reached those developmental ‘milestones’ at later ages than their less-accomplished national-class counterparts [16, 17].
  In addition, youth athletes who have an accelerated biological maturation, especially a younger onset of puberty and the growth spurt [18], and those born earlier within their age year (relative age effect, RAE [19, 20]) have a performance advantage during adolescence in many sports. However, this performance advantage diminishes or is even reversed by adulthood (e.g., [21–23]).
2. Furthermore, athletes differ individually regarding further potential factors, within both junior and senior age groups, including coaches and coaching, teammates, parental support, achievement motivation, their sports club, high school, or college, facilities, equipment, and demands external to sport, especially academics and vocation. These factors may all change over time and those changes over time may, again, differ individually in terms of occurrence, timing, speed, and magnitude of changes [24–30].
3. It is mathematically impossible for all successful juniors to become equally successful seniors. For example, world and continental junior championships are held biennially or annually in two to three junior age-groups in each sport (e.g., U19, U17, U15). The ages of participants at a senior international championship typically range across ten or more years (often from late teens to late 30s). Therefore, a generation of senior athletes competing at the same international championships may include former finalists and medalists from up to ~ 30 previous international junior championships who are now all competing for the same senior international finals and medals.
4. Finally, a related difficulty in studying precursors of senior success is that many youth athletes, including even highly successful juniors, withdraw from sports before adulthood, whether involuntarily (e.g., as a result of injury [31]) or voluntarily. It is difficult to quantify this phenomenon because studies have typically considered sport-specific dropout [31], though many dropouts from one sport continue on in another sport or begin a new sport [32–34].

Which view is supported empirically? Studies in the literature have investigated athletes’ longitudinal junior-to-senior performance development in terms of their junior and senior competition levels in their respective main sport. Prospective studies typically involved a junior sample at a defined junior competition level (e.g., competing at international junior championships) and determined how many of them later achieved an equivalent competition level at senior age (e.g., international senior championships). Retrospective studies typically involved a senior sample at a defined senior competition level (e.g., competing at international senior championships) and determined how many of them had competed at an equivalent competition level when they were juniors (e.g., international junior championships).

The evidence in the literature is mixed. Individual prospective studies have reported percentages of juniors who went on to achieve an equivalent competition level at senior age ranging from 0 to 68% [35, 36]. Similarly, individual retrospective studies have reported percentages of seniors who had achieved an equivalent competition level when they were juniors ranging from 2 to 100% [37, 38]. However, the studies varied in terms of junior age categories, junior and senior competition levels, sex, types of sports, and sample sizes (9 < *n* < 4456).

### Present Study

The present study aimed to establish more robust and generalizable findings via a systematic review and synthesis of findings. We considered three competition levels—competing at a national championship level, competing at an international championship level, and winning international medals—and addressed the following questions:

Question 1: How many junior athletes reach an equivalent competition level when they are senior athletes?

Question 2: How many senior athletes reached an equivalent competition level when they were junior athletes?

The proportion of athletes with equivalent junior and senior competition level for both prospective (Question 1) and retrospective studies (Question 2) is expressed as a percentage (number of athletes with equivalent junior and senior competition level within a sample/total number of athletes in the sample) and is hereafter labelled the ‘percentage with equivalent competition level’ (PECL). Together, the answers to Questions 1 and 2 provide an answer to our third question:

Question 3: To what extent are successful juniors and successful seniors one identical population or two disparate populations?

If they are largely one identical population, this suggests that junior success is indicative of senior success and, therefore, a high level of junior success is typically a prerequisite for a high level of senior success. Such a result would support theories of giftedness and the deliberate practice view, as well as the current system of talent promotion. By contrast, if successful juniors and successful seniors are largely two disparate populations, this suggests that junior success has limited relevance to senior success. Such a result would correspond to recent findings indicating that predictors of junior and senior success are different and partly opposite [16, 17, 39]. Further, such a result would counter theories of giftedness and the deliberate practice view, and call into question the current system of talent promotion.

## Methods

**Fig. 1:**
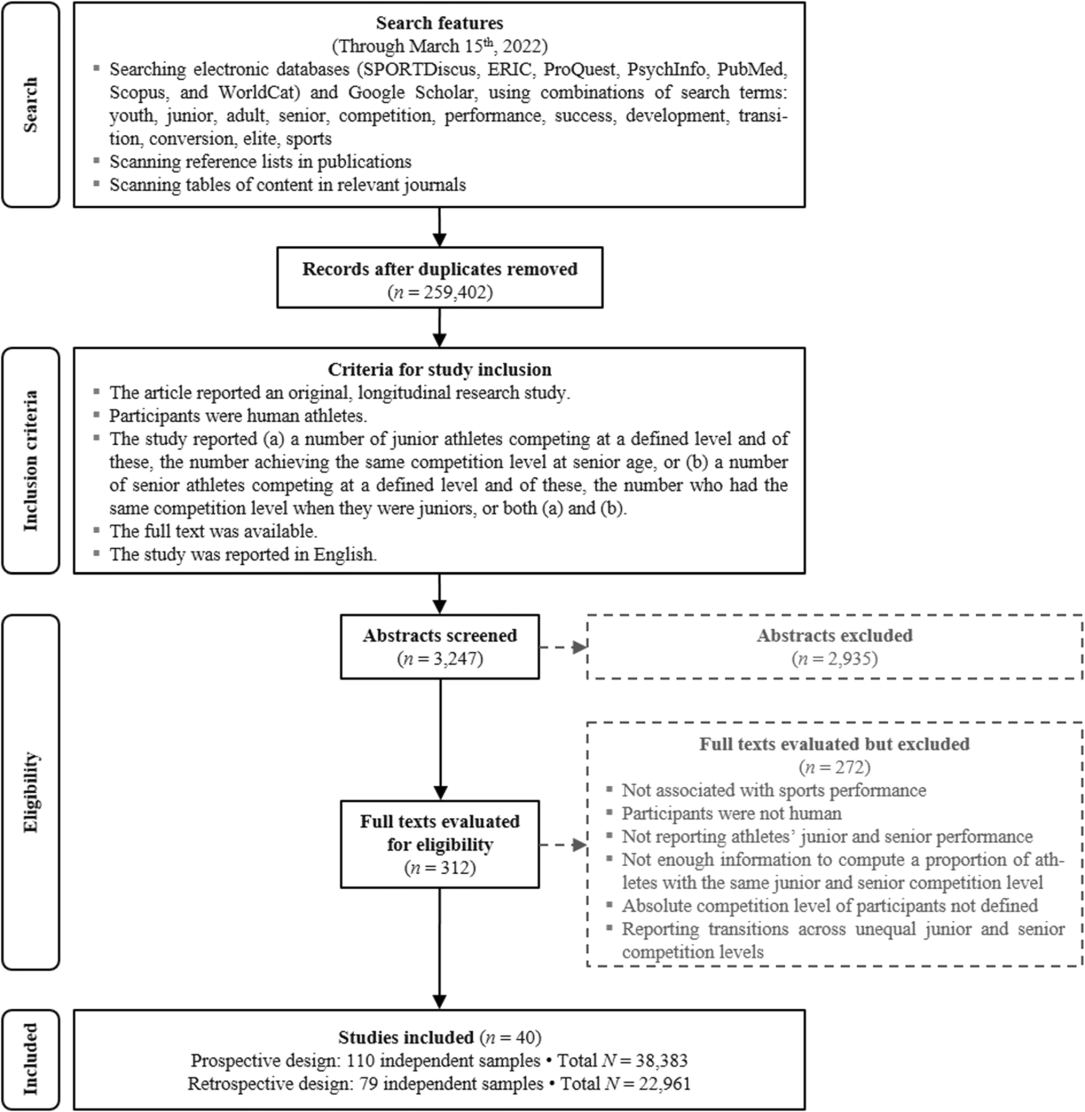
Flow diagram of the literature search and study coding

The study search and selection procedures were guided by the PRISMA 2020 statement (Preferred Reporting Items for Systematic Reviews and Meta-Analyses [40]; a PRISMA-P protocol was registered at https://osf.io/gck4a/). Figure 1 shows the flowchart of the major steps of the search and screening, which was conducted from January 27 through March 15, 2022.^1^

There were two complementary types of eligible studies. *Prospective* studies began with junior records, then found those athletes’ performance records when they were seniors. This approach could be biased by dropout. For junior athletes who withdrew from sports before senior age, we do not know how many would have been equally successful seniors had they continued competing. *Retrospective* studies are not subject to this limitation, because they begin with senior athlete records and then find those athletes’ performance records from when they were juniors. This approach captures those who became senior athletes, but likely yields higher PECL estimates due to possible survivorship bias. Thus, these two approaches offer complementary methodologies accounting for the type of potential bias imposed by the other.

### Sample

The search yielded a total of 189 study samples included in 40 study reports from 1995 to 2021. Each study was coded for (1) descriptive data, (2) publication status, (3) sample characteristics (age, sex, sport, country, competition level, junior age categories involved), (4) the total number of participants, and (5) the number of participants who had an equivalent competition level at junior and senior ages (i.e., competing at junior and senior national championships, at junior and senior international championships, or winning international junior and senior medals) (see Table S1 in the electronic supplementary material [ESM]).

Across studies, all sports of the Olympic Games, as well as Australian football, were represented. Many primary studies used comprehensive sampling such as including all participants at defined Olympic Games, all medalists at defined world championships, or all national team members over a defined number of seasons (see ESM, Table S1). Other studies involved samples of the members of a subset of youth sport academies or respondents to an athlete survey (including self-report of their competition level; for the high reliability of these self-reports, see [39, 41]). Tables 1 and 2 show characteristics of the total sample.

**Table 1:** Sample characteristics and subsample sizes of the prospective studies

Subsample	*N*
Year of study report	
Until 2009	3860
2010–2014	7766
2015–2021	26,757
Sex	
Male	23,570
Female	14,813
Individual vs team sports	
Individual sports (e.g., athletics, race cycling, swimming, tennis)	34,500
Team sports (e.g., basketball, handball, rugby, soccer)	3883
Types of sports by the task in competition^a^	
Cgs sports^b^: alpine skiing (72), athletics (10,896), race cycling (2840), swimming (16,170)	29,978
Game sports: basketball (61), handball (937), rugby (1325), soccer (1560), tennis (3898)	7781
Combat sport: taekwondo (624)	624
Region	
International samples (e.g., participants at junior world or continental championships)	32,188
National samples	
Western European countries	4664
Oceanian countries	1531
Junior competition level	
National level (national junior championships, national ranking top 20)	14,953
International level (junior world or continental championships, international ranking top 20)	21,495
International medal (medalists at junior world or continental championships)	1935
Junior age category^c^	
Junior A	25,656
Junior B	21,764
Junior C	5420
Junior D	0

^a^Analytical categorization of sports following [39]

^b^Cgs sports: sports where the performance is measured in centimetres, grams, or seconds

^c^Junior A: oldest junior age category within each sport, in most sports 17–18 or 18–19 years; Junior B: one age category below; Junior C: two age categories below; Junior D: three age categories below, in most sports 11–12 or 12–13 years. There were no available prospective studies for Junior D age

**Table 2:** Sample characteristics and subsample sizes of the retrospective studies

Subsample	*N*
Year of study report	
Until 2009	7355
2010–2014	2145
2015–2021	13,461
Sex	
Male	13,144
Female	9817
Individual vs team sports	
Individual sports (e.g., athletics, judo, race cycling, swimming, tennis)	15,587
Team sports (e.g., basketball, hockey, rugby, soccer, volleyball)	2383
Multi-sport samples (e.g., participants or medalists at Olympic Games)	4991
Types of sports by the task in competition^a^	
Cgs sports^b^: athletics (8444), bob/luge (29), canoe/kayak (192), ice speed skating (23), race cycling (2676), rowing (420), skiing (alpine, Nordic: 91), swimming (5042), triathlon (104), weightlifting (69)	17,090
Game sports: Australian rules football (911), badminton (102), baseball/softball (143), basketball (402), curling (11), field hockey (261), handball (235), ice hockey (17), rugby (388), soccer (588), table tennis (87), tennis (266), volleyball (238), water polo (168)	3817
Combat sports: boxing (97), fencing (109), judo (152), taekwondo (39), wrestling (330)	727
Artistic composition sports: gymnastics (artistic, rhythmic, trampoline: 306), figure skating (9), platform diving (102), synchronized swimming (57)	474
Other types of sports^c^: equestrian (133), modern pentathlon (55), sailing/windsurfing (277), shooting/archery (388)	853
Region	
International samples (e.g., participants at Olympic Games, world or continental championships)	18,587
National samples	
Western European countries	3196
Oceanian countries	1178
Senior competition level	
National level (national championships, national ranking top 20)	2600
International level (participants at Olympic Games, senior world or continental championships)	18,921
International medal (medalists at Olympic Games, senior world or continental championships)	1440
Junior age category^d^	
Junior A	22,462
Junior B	9025
Junior C	2930
Junior D	1005

^a^Analytical categorization of sports following [39]

^b^Cgs sports: sports where the performance is measured in centimetres, grams, or seconds

^c^Other types of sports: sports that meet none or various of the criteria of the aforementioned types of sports

^d^Junior A: oldest junior age category within each sport, in most sports 17–18 or 18–19 years; Junior B: one age category below; Junior C: two age categories below; Junior D: three age categories below, in most sports 11–12 or 12–13 years

#### Junior Age Categories

Junior age categories are defined by the international sport federation for each sport (e.g., U19, U17, U15). In most sports, the junior age limit is 18 or 19 years, but there are a few exceptions (e.g., female artistic and rhythmic gymnastics = 15 years, female swimming and male artistic gymnastics = 17 years, fencing and judo = 20 years). We used the official age groups of each sport.^2^

Labels for the different junior age groups differ by sport and country: e.g., U19, U17, U15, etc., ‘juniors,’ ‘youth,’ ‘cadets,’ ‘espoirs,’ ‘schoolboys,’ ‘schoolgirls,’ ‘cubs,’ ‘futures’. Throughout this report, we label the junior age categories as ‘Junior A’, ‘Junior B’, ‘Junior C’, and ‘Junior D’, with ‘Junior A’ being the oldest junior age group within each sport (in most sports, 17–18 or 18–19 years), ‘Junior B’ being the age category one younger than Junior A, ‘Junior C’ being two age categories below Junior A, and ‘Junior D’ being the youngest age group (mostly 11–12 or 12–13 years).

#### Performance Levels

Performance levels were defined by athletes’ competition levels, that is, athletes’ championship level (Olympic Games, senior or junior world, continental, or national championships) and placing (medalists, finalists, participants), or their placing in official international or national rankings. This approach allows us to include athletes’ performances across all types of sports.

We distinguished three competition levels at junior and senior age: national level: participants at national championships or top 20 in national rankings; international championship level: participants at the major international championships (Olympic Games, junior and senior world and continental championships) or top 20 in international rankings; and international medalists: medalists at Olympic Games or at junior or senior world or continental championships. Two slightly wider samples, the top 50 in World Athletics (formerly IAAF) ranking [42] and the top 25 in the European swimming (LEN) ranking [35], were considered international-level, rather than national-level samples, because their season-best jump heights or lengths and swim times corresponded to international championship participants.

Several studies additionally considered numbers of athletes achieving either the same level *or* up to one level below (e.g., junior international medalists → senior international medalists *or* finalists; junior international participants → senior international participants *or* national finalists). We considered whether additionally analyzing equivalent levels or one level below would yield supplementary information. However, differences between approaches were negligible (1.038 < odds ratio [OR] < 1.053; see Sect. 2.2 for the set significance criterion): prospective analyses 23.1% (95% confidence interval [CI] 22.6–23.5) versus 23.7% (95% CI 23.3–24.2, OR 1.039); retrospective analyses 30.6% (95% CI 30.0–31.2) vs 31.7% (95% CI 31.1–32.3, OR 1.053). Therefore, subsequent analyses refer to percentages of athletes achieving the same competition level (national, international, or international medalist) at junior and senior age.

It may be that a study examining how many athletes competing at a national junior championship went on to compete at a national senior championship might have included national championship athletes who qualified for an international championship. Similarly, participants at an international championship might have included medalists. Thus, when we report proportions of national- or international-level junior athletes who achieved the same competition level at senior age, these values may include a few athletes who achieved the same level and also one level higher. Likewise, reported proportions of national- or international-level senior athletes who had achieved an equivalent competition level when they were juniors may include a few athletes who had achieved the same level and one level higher. Given the number of participant places at the different levels, less than approximately 10% could have achieved one level higher.

In summary, we analyzed prospectively and retrospectively how many athletes reached an equivalent competition level at both junior and senior age.

#### Prospective Studies

One hundred ten independent samples, with a total of 38,383 athletes, 61.4% male and 38.6% female, were included in the prospective studies. Of these athletes, 21,495 competed at junior world and continental championships and 1935 were junior international medalists (see Table 1).

Since many studies considered multiple junior age categories, the 110 independent samples included a total of 151 PECL values: 93 for Junior A to senior, 36 for Junior B to senior, and 22 for Junior C to senior.

The primary studies either reported athletes’ ages as the sample mean and standard deviation or the minimum to maximum ages. Across studies, the sample-weighted mean age as a senior was 26.2 years, the sample-weighted mean minimum age as a senior was 20.0 years and the sample-weighted mean maximum age was 35.1 years.

Most athletes (83.9%) were from international samples (from multiple countries, e.g., the participants at international junior championships or the athletes listed in international junior rankings); the remaining athletes (16.1%) were from national samples from Western European or Oceanian countries (see Table 1). Data were collected from publicly available records (official championship results, ranking lists) for 38,373 athletes and in one study by interviews with 10 athletes.

#### Retrospective Studies

Seventy-nine independent samples, with a total of 22,961 athletes, 57.2% male and 42.8% female, were included in the retrospective studies. Of these athletes, 18,921 competed at Olympic Games and senior world or continental championships and 1440 were senior international medalists (see Table 2).

Since many studies considered multiple junior age categories, the 79 independent samples included a total of 188 PECL values: 76 for Junior A to senior, 46 for Junior B to senior, 57 for Junior C to senior, and 9 for Junior D to senior.

Across studies, the senior athletes’ sample-weighted mean age was 26.0 years, the sample-weighted mean minimum age was 20.9 years and the sample-weighted mean maximum age was 35.7 years. Most athletes (81.0%) were from international samples (from multiple countries, e.g., the participants at Olympic Games or world championships); the remaining athletes (19.0%) were from national samples from Western European and Oceanian countries (see Table 2). Data were collected from publicly available records (official championship results or ranking lists) for 21,580 athletes and by athlete surveys for 1381 athletes.

### Data Analysis

We used the PECL from each study to compute prevalence in the same manner as epidemiological prevalence: *x* ‘positive’ cases per a total of *n* cases, where ‘positive’ was defined here as achieving an equivalent competition level at junior and senior age.

We obtained the total number of participants, *n*, and the number of athletes who achieved an equivalent competition level at junior and senior age, *x*, from each study sample. Those numbers were then aggregated across study samples to establish *X*/*N*, separately for prospective and retrospective studies, for competition levels, and for junior age categories.

When *X*/*N* has been established from prospective and retrospective analyses, we can estimate the extent to which athletes achieving a defined competition level at junior and at senior age are the same or different athletes. This can be computed for each competition level and junior age category as follows:

$$\begin{aligned}&{\mathrm{Percentage}}_{\mathrm{identical}}\\&= \frac{X/{N}_{\mathrm{prospective}}}{1+\left(1- X/{N}_{\mathrm{retrospective}}\right)/X/{N}_{\mathrm{retrospective}} \times X/{N}_{\mathrm{prospective}}},\end{aligned}$$

or interchangeably:

$$\begin{aligned}&{\mathrm{Percentage}}_{\mathrm{identical}}\\&= \frac{X/{N}_{\mathrm{retrospective}}}{1+\left(1- X/{N}_{\mathrm{prospective}}\right)/X/{N}_{\mathrm{prospective}} \times X/{N}_{\mathrm{retrospective}}},\end{aligned}$$

where:

$${\mathrm{Percentage}}_{\mathrm{disparate}}= 1-{\mathrm{Percentage}}_{\mathrm{identical}}.$$

This formula thus estimates the extent to which successful juniors and successful seniors are one identical population or two disparate populations.

We investigated the question of whether prospective and retrospective *X*/*N* percentages (PECL) varied by sex, junior age category (Junior A–D), performance level (national, international championships, international medal), individual versus team sports, and publication status (published studies versus unpublished studies such as unpublished theses) by computing the odds ratio (OR) across the relevant subgroups. The conventional significance criterion for 2 × 2 contingency tables of *χ*^*2*^ > 3.841 and *p* < 0.05 was not appropriate for the present analyses (see [43, 44]). Because of the large sample sizes, *χ*^*2*^ > 3.841 and *p* < 0.05 would result from ORs as small as 1.041, corresponding to < 0.75% of the variance explained. For instance, in our dataset, a group difference of 10.3% versus 9.7%, OR 1.069, yields *χ*^*2*^ = 3.849, *p* < 0.05. Declaring effect sizes near zero as significant contradicts researchers’ intentions of signifying meaningful effects [43, 44]. Therefore, for the subgroup comparisons, we set our criterion for significance as an OR equivalent to explaining at least 1.0% of the variance (OR ≥ 1.437). In addition, we analyzed potential association of PECL with the time of each study by computing Pearson’s correlation between the PECL and the year of publication for each sample.

The 95% CI of each prospective and retrospective PECL is reported as the Agresti-Coull interval [45], as recommended for *n* > 40 [46].

### Quality Assessment and Risk of Bias

We appraised the quality of the primary studies using the Mixed Methods Appraisal Tool (MMAT) version for descriptive quantitative studies [47]. The MMAT assesses methodological quality criteria concerning sampling strategy, representativeness, validity of measurements, potential non-response bias, and appropriateness of statistical analyses. The assessment was performed on all studies by the first author and a random sample of 1/3 of the total studies (13 studies) was independently evaluated by the second author. Inter-rater reliability was excellent (Cohen’s *κ* = 0.97).

## Results

**Fig. 2:**
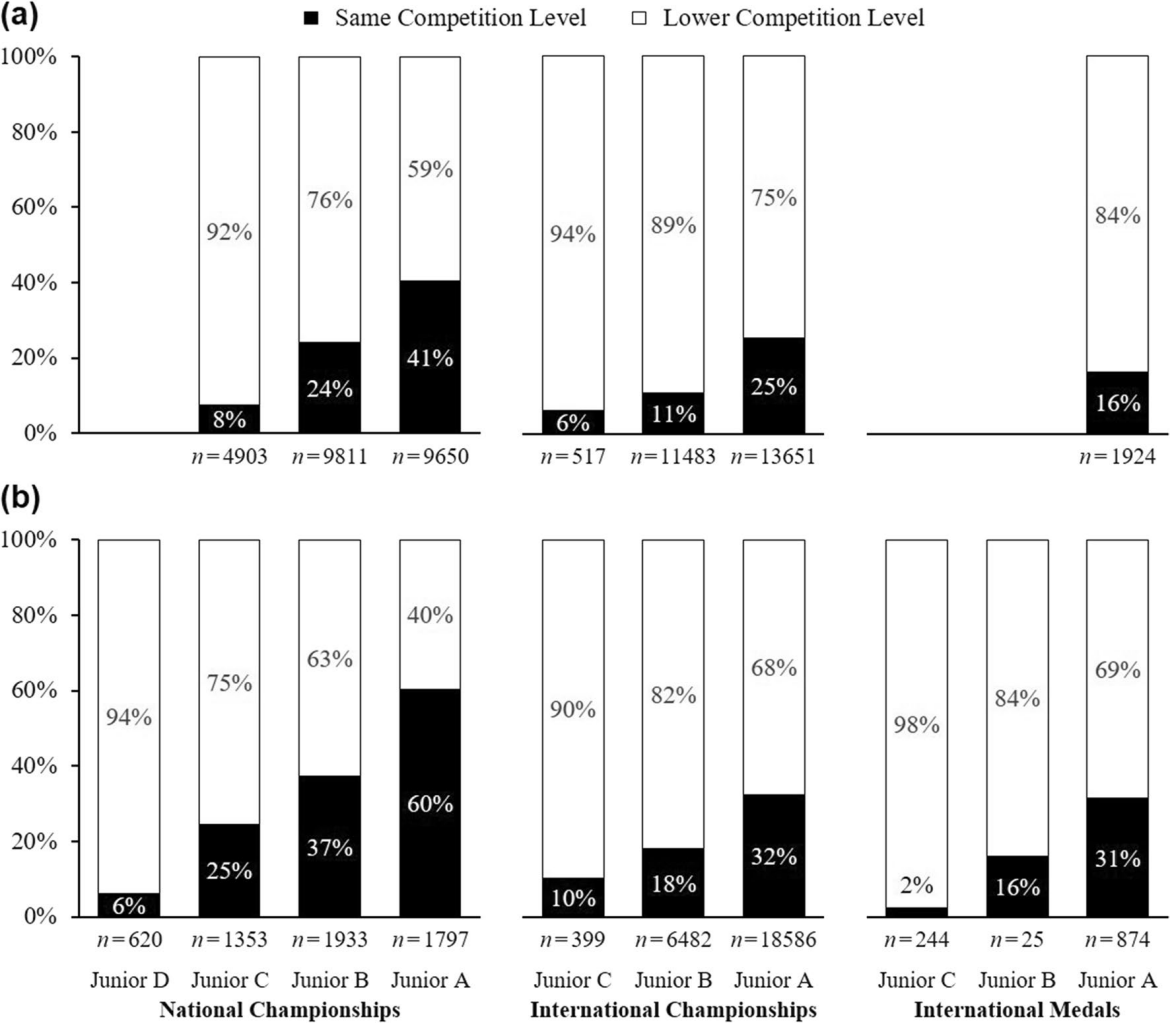
Percentages of athletes who achieved an equivalent competition level at junior and senior age. Top **a**: prospective analyses, percentage of junior athletes who achieved an equivalent (black) or a lower competition level (white) at senior age. Bottom **b**: retrospective analyses, percentage of senior athletes who had achieved an equivalent (black) or a lower competition level (white) when they were juniors. The numbers below each bar represent the number of athletes involved in each analysis. Junior A = oldest junior age category within each sport, in most sports 17–18 or 18–19 years; Junior B = one age category below; Junior C = two age categories below; Junior D = three age categories below, in most sports 11–12 or 12–13 years. The prospective studies included no data for international junior medalists at Junior B and Junior C ages and no analyses at any competition level for Junior D age. The 95% confidence intervals are presented in Tables 3 and 4

Figure 2 provides an overview of the central results. The upper panel (a) shows the percentage of junior athletes who achieved an equivalent competition level (black) versus a lower level (white) at senior age. The bottom panel (b) shows the percentage of senior athletes who had achieved an equivalent competition level (black) versus a lower level (white) when they were juniors.

**Table 3:** Comparisons of different competition levels within each junior age category

Junior age categories and competition levels	Percentage with equivalent junior and senior level				OR	Sig.
	Lower performers^a^		Higher performers^a^			
	Percent	95% CI	Percent	95% CI		
Prospective analyses^b^						
Junior C age						
National vs international	7.5	6.8–8.3	6.0	4.2–8.4	1.240	
Junior B age						
National vs international	24.2	23.3–25.0	10.8	10.3–11.4	2.622	*
Junior A age						
National vs international	40.5	39.6–41.5	25.3	24.6–26.0	2.014	*
National vs int. medal	40.5	39.6–41.5	16.3	14.6–18.0	3.511	*
International vs int. medal	25.3	24.6–26.0	16.3	14.6–18.0	1.743	*
Retrospective analyses						
Junior C age						
National vs international	24.6	22.4–27.0	10.3	7.6–13.7	2.851	*
National vs int. medal	24.6	22.4–27.0	2.5	1.0–5.4	12.950	*
International vs int. medal	10.3	7.6–13.7	2.5	1.0–5.4	4.543	*
Junior B age						
National vs international	37.2	35.1–39.4	18.0	17.1–19.0	2.689	*
National vs int. medal	37.2	35.1–39.4	16.0	5.8–35.3	3.109	*
International vs int. medal	18.0	17.1–19.0	16.0	5.8–35.3	1.156	
Junior A age						
National vs international	60.2	57.9–62.5	32.5	31.9–33.2	3.227	*
National vs int. medal	60.2	57.9–62.5	31.4	28.4–34.5	3.314	*
International vs int. medal	32.5	31.9–33.2	31.4	28.4–34.5	1.027	

*CI* confidence interval reported as Agresti-Coull interval. *Int.* international, *OR* odds ratio, *Sig.* significance

*Subgroup differences are considered significant when OR ≥ 1.437 (i.e., ≥ 1% variance explained)

^a^Lower and higher performers refer to the competition levels defined in the pre-column

^b^The prospective analyses included no data for international junior medalists at Junior B and Junior C ages

**Table 4:** Comparisons of different junior age categories within each competition level

Competition levels and junior age categories	Percentage with equivalent junior and senior level				OR	Sig.
	Younger age group^a^		Older age group^a^			
	Percent	95% CI	Percent	95% CI		
Prospective analyses^b^						
International level						
Junior C vs Junior B	6.0	4.2–8.4	10.8	10.3–11.4	1.905	*
Junior C vs Junior A	6.0	4.2–8.4	25.3	24.6–26.0	5.499	*
Junior B vs Junior A	10.8	10.3–11.4	25.3	24.6–26.0	2.788	*
National level						
Junior C vs Junior B	7.5	6.8–8.3	24.2	23.3–25.0	3.902	*
Junior C vs Junior A	7.5	6.8–8.3	40.5	39.6–41.5	8.356	*
Junior B vs Junior A	24.2	23.3–25.0	40.5	39.6–41.5	2.141	*
Retrospective analyses						
International medals						
Junior C vs Junior B	2.5	1.0–5.4	16.0	5.8–35.3	7.556	*
Junior C vs Junior A	2.5	1.0–5.4	31.4	28.4–34.5	18.114	*
Junior B vs Junior A	16.0	5.8–35.3	31.4	28.4–34.5	2.398	*
International level						
Junior C vs Junior B	10.3	7.6–13.7	18.0	17.1–19.0	1.932	*
Junior C vs Junior A	10.3	7.6–13.7	32.5	31.9–33.2	4.204	*
Junior B vs Junior A	18.0	17.1–19.0	32.5	31.9–33.2	2.186	*
National level						
Junior D vs Junior C	6.1	4.5–8.3	24.6	22.4–27.0	5.000	*
Junior D vs Junior B	6.1	4.5–8.3	37.2	35.1–39.4	9.071	*
Junior D vs Junior A	6.1	4.5–8.3	60.2	57.9–62.5	23.177	*
Junior C vs Junior B	24.6	22.4–27.0	37.2	35.1–39.4	1.814	*
Junior C vs Junior A	24.6	22.4–27.0	60.2	57.9–62.5	4.635	*
Junior B vs Junior A	37.2	35.1–39.4	60.2	57.9–62.5	2.555	*

*CI* confidence interval reported as Agresti-Coull interval. *OR* odds ratio, *Sig.* significance

*Subgroup differences are considered significant when OR ≥ 1.437 (i.e., ≥ 1% variance explained)

^a^Younger and older age group refers to the junior age categories defined in the pre-column

^b^The prospective studies included no data for international junior medalists at Junior B and Junior C age and no analyses at all for Junior D age

Very few successful junior athletes went on to reach an equivalent competition level at later senior age. For example, only 16.3% of junior international medalists at Junior A age became senior international medalists, whereas 83.7% did not; 6.0%, 10.8%, and 25.3% of international-level juniors at Junior C, B, and A age, respectively, became international-level seniors, whereas 94.0%, 89.2%, and 74.7% did not; and 7.5%, 24.2%, and 40.5% of national-level juniors at Junior C, B, and A age, respectively, became national-level seniors, whereas 92.5%, 75.8%, and 59.5% did not (see Fig. 2, Panel a).

Likewise, very few successful senior athletes had achieved equivalent competition levels when they were juniors. Only 2.5%, 16.0%, and 31.4% of all senior international medalists had been junior international medalists at Junior C, B, and A age, respectively, whereas 97.5%, 84.0%, and 68.6% had not; 10.3%, 18.0%, and 32.5% of all international-level seniors had been international-level juniors at Junior C, B, and A age, respectively, whereas 89.7%, 82.0%, and 67.5% had not; and 6.1%, 24.6%, 37.2%, and 60.2% of all national-level seniors had been national-level juniors at Junior D, C, B, and A age, respectively, whereas 93.9%, 75,4%, 62.8%, and 39.8% had not (see Fig. 2, Panel b).

Findings were also consistent at the very highest performance level: senior international gold medalists. In six retrospective studies, 584 Olympic and world championship gold medalists were identified (not shown separately in Fig. 2); 2.9% had been international junior gold medalists at Junior C age, whereas 97.1% had not, and 28.1% had been international junior gold medalists at Junior A age, whereas 71.9% had not. (There were no available data for international junior gold medalists at Junior B age.)

**Fig. 3:**
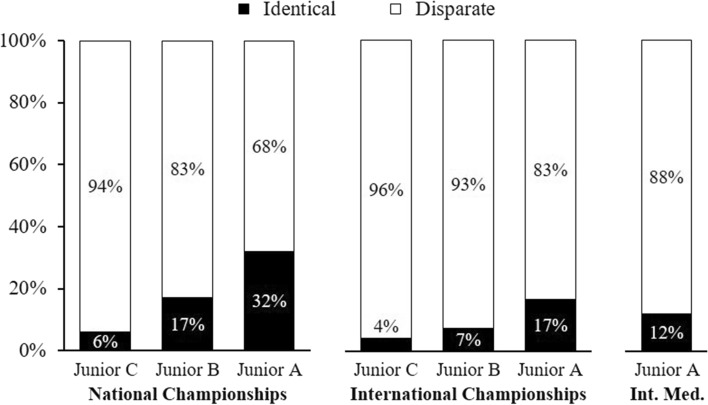
The extent to which successful juniors and successful seniors are one identical population (black) or two disparate populations (white). *Int. Med.* international medals

Combining the prospective and retrospective analyses enabled the calculation of an estimate quantifying the extent to which successful juniors and successful seniors are one identical population or two disparate populations. The results are presented in Fig. 3. Consistently across performance levels and junior age categories, the successful juniors and successful seniors are largely two disparate populations. For instance, the groups with the smallest overlap, international-level athletes at Junior C and international-level athletes at senior age, were 3.9% identical and 96.1% disparate. The groups with the largest overlap, national-level athletes at Junior A and national-level athletes at senior age, were 32.0% identical and 68.0% disparate (see Fig. 3).

In the subsequent Sects. 3.1–3.4 and 3.6, we report comparisons of the PECL between defined subsamples and whether the OR exceeded the critical value of 1.437 set for statistical significance (see Sect. 2.2).

### Variation by Sex

The results did not differ significantly by sex overall. In the prospective analyses, the PECL was 18.5% (95% CI 18.0–19.1) for males and 21.9% (95% CI 21.2–22.7) for females (OR 1.234). In the retrospective analyses, the PECL was 29.8% (95% CI 28.8–30.8) for males and 30.3% (95% CI 28.9–31.7) for females (OR 1.025).

### Variation by Competition Level

Whether analyzed prospectively or retrospectively, the PECL was generally smaller at higher than lower competition levels (see Table 3). Though percentages varied by performance level, they were all low, with most junior athletes achieving lower competition levels at senior age and most senior athletes having achieved lower competition levels when they were juniors.

### Variation by Junior Age Category

Whether analyzed prospectively or retrospectively, the PECL was generally smaller among younger than older junior age categories (see Table 4). Percentages varied by junior age category, but they were generally low.

### Variation by Individual Versus Team Sports

Differences between individual and team sports were inconclusive. In prospective analyses, the PECL was significantly larger in individual than team sports for Junior A age (34.9% vs 14.4%, OR 3.195), the difference was non-significant for Junior B age (16.7% vs 12.9%, OR 1.354), and the PECL was significantly smaller in individual than team sports for Junior C age (6.2% vs 11.0%, OR 1.869). In retrospective analyses, the PECL was significantly smaller in individual than team sports for Junior A (30.2% vs 49.9%, OR 2.309) and Junior B ages (20.5% vs 32.4%, OR 1.857), respectively, the difference was non-significant for Junior C age (19.8% vs 17.1%, OR 1.195), and the PECL was significantly larger in individual than team sports for Junior D age (6.4% vs 0.0%, OR 3.378 [Haldane-corrected]).

### Variation by Year of Publication

Among prospective studies, the correlation between the PECL and the year of publication was *r* = 0.05 (*p* = 0.526) and among retrospective studies, the correlation was *r* = – 0.18 (*p* = 0.013). The findings suggest that the year of the studies did not substantially predict the PECL. If anything, the percentage of successful senior athletes who had competed at an equivalent level when they were juniors has slightly diminished. Correspondingly, the percentage of successful seniors who had previously competed at lower levels when they were juniors has slightly increased across the observation period (1995–2021).

### Variation by Publication Status

Of the total 40 study reports, 29 were published (11 prospective, 8 retrospective, and 10 reporting both prospective and retrospective accounts) and eleven unpublished (e.g., unpublished theses; 2 prospective, 7 retrospective, and 2 reporting both). Among prospective analyses, the PECL was significantly larger in published than in unpublished studies overall (33.8%, 95% CI 33.2–34.4 vs 23.7%, 95% CI 21.7–25.8, OR 1.641). The finding suggests that prospective studies reporting higher rates of successful junior-to-senior transitions were more likely to be published than those reporting lower rates. This was not the case for retrospective analyses; the PECL did not significantly differ between published and unpublished study reports (30.2%, 95% CI 29.6–30.8, vs 35.0%, 95% CI 32.8–37.3, OR 1.246).

### Quality Assessment and Risk of Bias

All primary studies had a high methodological quality and the risk of bias was generally low. See the ESM, Table S2, for details.

## Discussion

The study investigated the percentage of junior athletes who went on to achieve an equivalent competition level at senior age in their respective main sport, the percentage of senior athletes who had competed at an equivalent level when they were juniors, and the extent to which successful juniors and successful seniors are one identical or two disparate populations. Analyses involved 189 study samples including 38,383 athletes in prospective and 22,961 athletes in retrospective studies. All athletes competed at national championship or international championship levels at junior or senior age, or both, and were from a wide range of sports and both sexes.

Three central findings emerged:

1. Few junior athletes go on to achieve an equivalent competition level when they are seniors; most elite (national-level or higher) junior athletes achieve lower competition levels at senior age. Likewise, few senior athletes achieved an equivalent competition level when they were juniors; most elite senior athletes achieved lower competition levels at a junior age.
2. Successful juniors and successful seniors are largely two disparate populations.
3. The percentages of athletes achieving equivalent competition levels at junior and senior age (PECL) were generally the smallest among the highest competition levels and the youngest junior age categories.

The findings were robust across competition levels, junior age categories, male and female athletes, individual and team sports, prospective and retrospective analyses, and from the 1990s to the 2020s. There was one case, the lowest senior competition level, national senior championships, where 60.2% of national-level senior competitors had been national-level juniors in the oldest junior age category, Junior A. The other 23 analyses (see Figs. 2, 3) confirmed that successful juniors and successful seniors are largely two disparate populations.

The findings are consistent with studies in the literature reporting low rates of successful transitions from high school sports via NCAA conferences to professional leagues [48], as well as high rates of annual athlete turnover in sport federations’ youth squads and selection teams and in youth sport academies [49–51]. In addition, the present results are consistent with the meta-analytic finding [16, 17] that higher-performing junior athletes reach developmental performance-related ‘milestones’ (first national championships, first international championships) at a younger age than lower-performing juniors, whereas senior world-class athletes had reached those developmental ‘milestones’ at later ages than their senior national-class counterparts.

### Theoretical Implications

The present findings counter traditional theories of giftedness and expertise [1–4]. Both the giftedness and the deliberate practice hypotheses emphasize the importance of a high level of youth performance, although peak performance is typically achieved later in adulthood. Both hypotheses rest on the presuppositions that (1) successful juniors and successful seniors are largely one identical population, since (2) youth performance itself is a predictor of later, adult performance, and accordingly, (3) early youth performance and later adult performance are predicted by the same factors.

The present findings counter all three assumptions. The first assumption—that successful juniors and successful seniors are largely one identical population—has been falsified by the present result that successful juniors and successful seniors are largely two disparate populations. Regarding the second and third assumptions—that youth performance itself is a predictor of later, adult performance and that early youth performance and later adult performance are predicted by the same factors—our finding of the minimal amount of overlap of the populations of successful juniors and successful seniors implies that junior performance cannot be a strong predictor of senior performance. Accordingly, junior and senior performance cannot be predicted by the same factors. The third assumption is also countered by the findings that most of the highest-performing juniors do not go on to be among the highest-performing seniors and that most of the highest-performing seniors had performed below the highest-performing peers at junior age. These results imply that the highest-performing seniors had greater long-term performance improvement from junior to senior age than the highest performing juniors had. By inference, early junior performance and subsequent performance improvement are predicted by different factors.

Relatedly, recent meta-analyses have suggested that participation patterns that facilitate early junior performance hamper long-term senior performance, while participation patterns that facilitate long-term senior performance are associated with reduced junior performance ([16, 17]; see Sect. 1). Furthermore, extensive childhood/adolescent specialized practice is a predictor of early junior performance, but also of premature dropout [31].

Other factors also likely play a role in who is successful as a junior that may or may not translate to senior success. Performance develops through the interaction of the task, the person, and the environment during both junior and senior age [52]. Characteristics of the task (e.g., skill acquisition, movement solutions [52]), of the person (e.g., biological maturation, achievement motivation [18, 28]) and of the environment (e.g., coaching, single- or multi-sport engagement, parental support, demands from academics or vocation, etc. [16, 17, 24–27, 29, 30]) may all differ between athletes, may change over time, and the changes over time may differ between athletes. This may all contribute to the heterogeneity of the performance development across individual athletes. In addition, many athletes withdraw from a sport before adulthood [31]. However, the magnitude of general sport dropout of high-performing athletes is widely unknown [16, 17, 32–34].

**Fig. 4:**
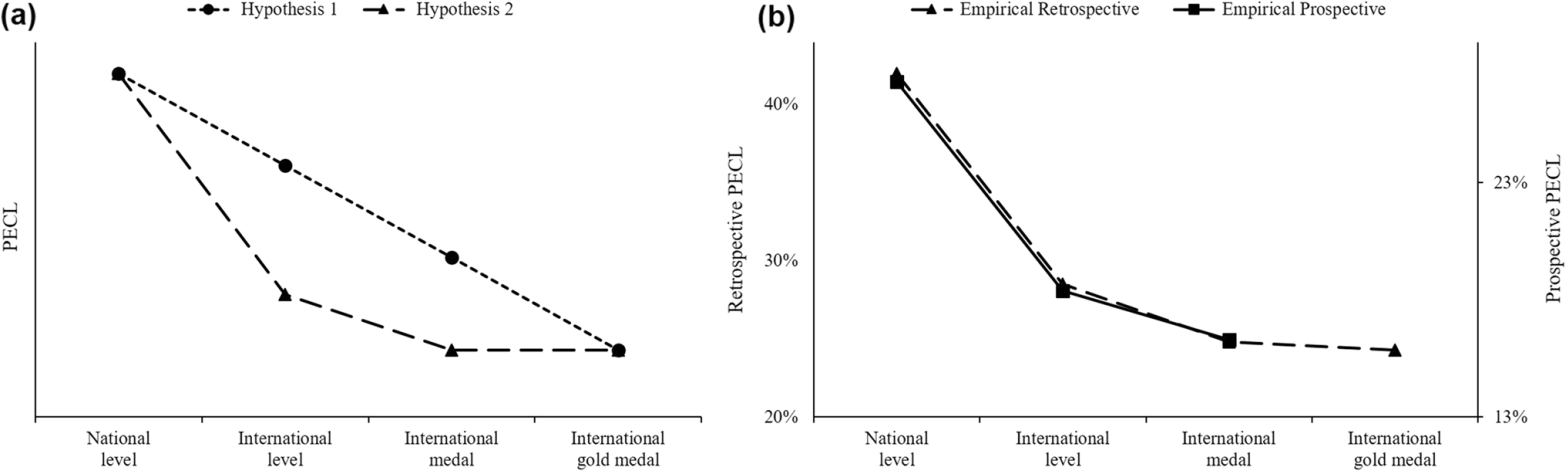
Proportions of athletes who achieve an equivalent competition level at junior and senior age (PECL), broken down across different competition levels. Panel (**a**) Schematic illustration of expected PECL according to explanatory hypotheses 1 and 2 (see main text). Panel (**b**) PECL revealed by the present empirical results (overall data across Junior A to C age for each competition level). Prospective studies included no junior and senior international gold medalists

Our finding that the PECL was smaller among higher performance levels also has important theoretical implications. There are two plausible explanatory hypotheses. Hypothesis 1 is based on the premise that the probability of achieving a higher competition level is smaller than the probability of achieving a lower competition level. The combination of lower probabilities (of achieving a higher competition level) at two time points—junior and senior age—leads to smaller proportions of athletes achieving the same competition level at both time points, and vice versa. Thus, according to this hypothesis, the PECL would decrease roughly linearly across increasing competition levels. See Fig. 4, Panel a.

Hypothesis 2 is based on the finding that the differences in predictors of junior and senior performance are more pronounced for senior world-class performance (international top ten) than for lower senior performance levels [16, 17]. According to this hypothesis, proportions of athletes achieving the national competition level at junior and senior ages will be greater than for athletes who place in the top ten at international championships at junior and senior ages. The competition levels defined in the present study widely match the definition of national- and world-class level in [16, 17]. There is a small deviation, in that in the present study about 20% of the international championship participants placed below the top ten.

According to Hypothesis 2, it is plausible to expect that the PECL will be distinctively greater for national level than for each of the higher, international levels—international championship participation, international medals, and international gold medals—whereas the differences within the latter three, international levels will be small. Since not all international championship participants achieved international top ten placings, the PECL may be slightly larger than among international medalists and gold medalists. In sum, according to this hypothesis, the PECL would decrease sharply from national to international level and then flatten. See Fig. 4, Panel a.

In summary, Hypothesis 1 suggests that smaller or larger probabilities of higher or lower competition levels are combined across two time points, whereas Hypothesis 2 suggests varying differences in predictors of junior and senior performance across competition levels. Figure 4, Panel b, shows the corresponding empirical data from prospective and retrospective analyses. Overall, the PECL was significantly larger for national level than all higher, international competition levels (1.816 < OR < 2.254), but did not significantly differ among the higher, international levels (1.028 < OR < 1.241; see Fig. 4, Panel b). Thus, the results provide stronger support for the second than the first explanatory hypothesis.

### Practical Implications

There are several practical and policy implications of the results of this study.

1. Most of the highest-performing seniors had a lower performance level at junior age than the highest-performing juniors, and, by inference, had greater long-term performance improvement through subsequent years. Thus, to improve athletes’ long-term senior performance, youth training strategies should primarily focus on the expansion of youth athletes’ potential for future long-term performance improvement through adulthood, rather than primarily seeking to accelerate their short-term junior performance.
2. The present findings suggest that current selection strategies for youth talent promotion programs—where the highest-performing youth athletes are preferably selected—are misguided. When national sport systems select and focus their resources on the highest junior performers (e.g., [6–8, 12]), most of the selected youth athletes will not become senior elite athletes, while most of the youth athletes who will be senior elite athletes in the future are dismissed. In addition, when selection criteria for talent promotion programs, as well as for sport scholarships, include youth athletes’ current junior performance, this may have a ‘radiating’ effect, in that it stimulates all those seeking admission to these programs—youth athletes, coaches, and parents—to attempt to accelerate youth athletes’ adolescent performance [50]. Instead, the goal should be to identify which of the juniors performing below their highest-performing peers are the ones who have the greatest potential for future multi-year performance improvement.
3. Relatedly, performance within junior age is not a sensible criterion for the evaluation of talent promotion programs or of youth coaches in general. When they are evaluated by current junior successes, this will stimulate attempts to select the most advanced youth athletes and to reinforce further acceleration of their adolescent performance development. This may expand youth athletes’ costs (e.g., their time and body [14, 33]) and risks (e.g., injury [53]), but may hamper their long-term sustainable development towards senior high performance [16, 17].
4. While the present findings do not generally speak against international junior championships, festivals, and circuits per se, their value should be put into perspective. Participation in international junior championships may provide unique life experiences, learning opportunities, practice of cross-cultural peaceful encounters, and international friendships for the youth athletes. However, viewing the value of participation in international junior competitions as a precursor of later participation in international senior competitions is clearly at odds with the empirical evidence.

Finally, two ethical issues should be considered. First, when talent promotion programs claim to select the youth athletes with the greatest future potential, as mentioned above, athletes’ current junior performance is neither a fair nor a sensible selection criterion. Second, in view of the minimal probability to become a successful senior athlete, the increased costs and risks imposed on the participants in talent promotion programs are difficult to reconcile with adults’ responsibility for youth athletes’ development and wellbeing within and outside of sports. The specialized training is expanded and the programs impose additional time demands on the youth athlete in terms of additional competitions, transit times, and participation in athlete services [15, 33]. Therefore, the youth athlete’s risks of future overuse injuries are increased (e.g., [53]) and at the same time, their opportunity costs (i.e., the lost benefit of foregone other activities) are magnified by reducing time with family, friends, other hobbies, and, most notably, educational activities and outcomes ([14], for a review). These increased costs and risks are imposed on all the selected youth athletes, the few who become successful senior athletes and equally so on the many who do not.

The issue is exacerbated because the question of whether the measures of talent promotion programs actually improve the youth athletes’ later senior performance is widely unstudied. However, what studies have shown is that—consistent with the present findings—a particularly young involvement in talent promotion programs—as well as excess childhood/adolescent specialized training and an early achievement of performance-related ‘milestones’—are all negatively correlated with senior world-class success (for reviews, see [14, 16, 17]).

### Methodological Considerations

The study has several strengths, such as a large international sample from a wide range of sports, considering different competition levels and junior age categories, a high methodological quality of primary studies, and the combination of prospective and retrospective designs. But several limitations should be acknowledged. First, the study is descriptive and does not speak to causal processes underlying more or less successful junior-to-senior transitions. Second, male samples, national samples from Western European and Oceanian countries, and samples from the sports of the Olympic Games, especially individual sports, were over-represented. Third, all athletes competed at a national or international level at either junior or senior age, or both age groups. It may be that proportions of successful junior-to-senior transitions differ at lower performance levels or among more heterogeneous samples. Finally, although we used multiple databases, as in any systematic review, bias of availability, country, and language is possible.

### Future Directions

Researchers should seek to extend investigations to populations that are under-represented in present research, especially females, sports other than those of the Olympic Games, Paralympic sports, team sports, and national samples from countries outside Western Europe and Oceania. Future investigations may complement the present approach by synthesizing findings that quantify the extent to which individual differences in junior performance explain individual differences in senior performance. Furthermore, it will be of particular interest to scrutinize indicators identifying who of the juniors performing below their highest-performing peers are those with the greatest long-term future potential to become senior elite athletes.

On a final note, the fact that successful juniors and successful seniors are largely two disparate populations indicates that theory development of expertise and giftedness should not extrapolate from junior-level performers (such as [1, 3, 54, 55]), as this leads to incorrect and misleading conclusions.

## Supplementary Information

Below is the link to the electronic supplementary material.

Supplementary file1 (XLSX 62 KB)

Supplementary file2 (DOCX 33 KB)

Supplementary file3 (XLSX 12 KB)

## Footnotes

1 This study search was independent from [16, 17] and established a separate data set.

2 When primary studies included junior athletes who competed at senior championships while still within junior age and achieved the same competition level in the senior as in the junior championships, these were registered as having achieved an equivalent junior and senior competition level.

## Declarations

### Data availability

The original data are freely available at https://osf.io/mabvu/.
