# Supplementary material for: Quantifying the Extent to Which Successful Juniors and Successful Seniors are Two Disparate Populations: A Systematic Review and Synthesis of Findings
Source: Sports Med. 2023 Apr 6;53(6):1201–17. doi: 10.1007/s40279-023-01840-1 (PMC10185603; doi:10.1007/s40279-023-01840-1)
Supplement: Supplementary file 2 — Supplementary file2 (DOCX 33 KB) [file 40279_2023_1840_MOESM2_ESM.docx]

**References** (studies included in the systematic review and synthesis)

Barkell, J., O’Connor, D., & Cotton, W. (2013). An examination of the progression from national schoolboy to senior representation in Australian rugby union. *University of Sydney Papers in Human Movement, Health and Coach Education, 2,* 1-16. <https://www.academia.edu/download/42253665/An_examination_of_the_progression_from_n20160206-14055-3ljlg7.pdf>

Barreiros, A. N., & Fonseca A. M. (2012). A retrospective analysis of Portuguese elite athletes’ involvement in international competitions. *International Journal of Sports Science & Coaching 7(3),* 593-600. [https://doi.org/10.1260/1747-9541.7.3.593](https://doi.org/10.1260%2F1747-9541.7.3.593)

Barth, M. (2019). Analysing international senior success in alpine ski racing: Success concentration and predictability. Unpublished manuscript, University of Innsbruck.

Bjoerndal, C. T., Luteberget, L. S., & Holm, S. (2018). The relationship between early and senior level participation in international women’s and men’s handball. *Journal of Human Kinetics, 63(1),* 73-84. <https://doi.org/10.2478/hukin-2018-0008>

Boccia, G., Moisè, P., Franceschi, A., et al. (2017). Career performance trajectories in track and field jumping events from youth to senior success: The importance of learning and development. *PLoS ONE, 12(1),* e0170744. <https://doi.org/10.1371/journalpone.0170744>

Boccia, G., Brustio, P. R., Moisè, P., et al. (2019). Elite national athletes reach their peak performance later than non-elite in sprints and throwing events. *Journal of Science and Medicine in Sport, 22(3),* 342-347. <https://doi.org/10.1016/j.jsams.2018.08.011>

Boccia, G., Cardinale, M., & Brustio, P. R. (2021). Performance progression of elite jumpers: Early performances do not predict later success. *Scandinavian Journal of Medicine & Science in Sports, 31(1),* 132-139. <https://doi.org/10.1111/sms.13819>

Brouwers, J., De Boscher, V., & Sotiriadou, P. (2012). An examination of the importance of performances in youth and junior competition as an indicator of later success in tennis. *Sport Management Review, 15(4),* 461-475. <https://doi.org/10.1016/j.smr.2012.05.002>

Brustio, P. R., Cardinale, M., Lupo, C., Varalda, M., De Pasquale, P., & Boccia, G. (2021). Being a top swimmer during the early career is not a prerequisite for success: A study on sprinter strokes. *Journal of Science and Medicine in Sport, 24(12),* 1272-1277. <https://doi.org/10.1016/j.jsams.2021.05.015>

Carazo-Vargas, P., & Moncada-Jiménez, J. (2014). Successful youth performance does not relate to future senior performance in elite taekwondo competitors. *International Journal of Physical Education, Fitness and Sports, 3(4),* 11-17. <https://doi.org/>[10.26524/1442](https://doi.org/10.26524/1442)

Cripps, A., Hopper, L. S., Joyce, C., & Veale, J. (2015). Pathway efficiency and relative age in the Australian Football League talent pathway. *Talent Development and Excellence, 7(1),* 3-11. <http://www.iratde.org/journal/issues/119-2015-2>

Drew, K. (2020). Investigating the junior-to-senior transition in sport: Interventions to support the transitional process. Thesis (PhD), John Moores University Liverpool. <http://researchonline.ljmu.ac.uk/id/eprint/13008/1/2020Drewphd.pdf>

Grund, M., & Ritzdorf, W. (2006). From talent to elite athlete: A study of the performance development of the finalists at the 1999 IAAF world youth championships. *New Studies in Athletics, 21(2),* 43-55. <https://www.worldathletics.org/nsa/article/filter?&year=2006&volume=21&issue=2>

Güllich, A. (2014a). Many roads lead to Rome – developmental paths to Olympic gold in men’s field hockey. *European Journal of Sport Science, 14(8),* 763-771 (unpublished data from the study). <https://doi.org/10.1080/17461391.2014.905983>

Güllich, A. (2014b). Selection, de-selection and progression in German football talent promotion. *European Journal of Sport Science*, *14*(6), 530–537. <https://doi.org/10.1080/17461391.2013.858371>

Güllich, A. (2018). Sport-specific and non-specific practice of strong and weak responders in junior and senior elite athletics – a matched-pairs analysis. *Journal of Sports Sciences, 36(19),* 2256-2264 (unpublished data from the study). <https://doi.org/10.1080/02640414.2018.1449089>

Güllich, A. (2019). “Macro-structure” of developmental participation histories and “micro-structure” of practice of German female world-class and national-class football players. *Journal of Sports Sciences, 37(12),* 1347-1355 (unpublished data from the study). <https://doi.org/10.1080/02640414.2018.1558744>

Güllich A, Emrich E. Considering long-term sustainability in the development of world class success (unpublished data from the study). *Eur J Sport Sci. 2014;14 Suppl 1*:S383-S397. <https://doi.org/10.1080/17461391.2012.706320>.

Güllich, A., Seiler, S., & Emrich, E. (2009). Training methods and intensity distribution of young world-class rowers. *International Journal of Sports Physiology and Performance, 4(4),* 448-460. <https://doi.org/10.1123/ijspp.4.4.448>

Hardy L, Laing S, Barlow M, Kincheva L, Evans L, Rees T, et al. *A comparison of the biographies of GB serial medal and non-medalling Olympic athletes.* London: UK Sport; 2013.

Hollings, S.C., & Hume, P.A. (2010). Is Success at the world junior athletics championships a prerequisite for success at world senior championships or Olympic Games? - Prospective and retrospective analyses. *New Studies in Athletics, 25(2),* 65-77. <https://www.worldathletics.org/download/downloadnsa?filename=fdc6e534-588b-4f55-9705-b1f6c01117d9.pdf&urlslug=is-success-at-the-iaaf-world-junior-athletics>

Hollings, S. C., & Hume, P. A. (2011). Progression of New Zealand and Australian world junior championship competitors to senior representation, *New Studies in Athletics, 26(3/4),* 127-135. <https://www.worldathletics.org/download/downloadnsa?filename=aa98c71d-a533-4998-b870-307824eba8ab.pdf&urlslug=progression-of-elite-new-zealand-and-australi>

Hornig, M., Aust, F., & Güllich, A. (2016). Practice and play in the development of German top-level professional football players (unpublished data from the study). *European Journal of Sport Science, 16(1),* 96-105. <https://doi.org/10.1080/17461391.2014.982204>

Kalén, A., Pérez-Ferreirós, A., Rey, E., & Padrón-Cabo, A. (2017). Senior and youth national team competitive experience: influence on player and team performance in European basketball championships. *International Journal of Performance Analysis in Sport, 17(6),* 832-847. <https://doi.org/10.1080/24748668.2017.1405610>

Kearney, P. E., & Hayes, P. R. (2018). Excelling at youth level in competitive track and field athletics is not a prerequisite for later success. *Journal of Sports Sciences, 36(21),* 2502-2509. <https://doi.org/10.1080/02640414.2018.1465724>

Kristiansen, E., & Stensrud, T. (2020). Talent development in a longitudinal perspective: Elite female handball players within a sport school system. *Translational Sports Medicine, 3(4),* 364-373. <https://doi.org/10.1002/tsm2.143>

Latorre-Roman, P. A., Pinillos, F. G., & Robles, J. L. (2018). Early sport dropout: High performance in early years in young athletes is not related with later success. *Retos, 33,* 210-212.

Leyhr, D., Kelava, A., Raabe, J., & Höner, O. (2018). Longitudinal motor performance development in early adolescence and ist relationship to adult success: An 8-year prospective study of highly talented soccer players. *PLoS ONE, 13(5),* e0196324. <https://doi.org/10.1371/journal.pone.0196324>

Pizzuto, F., Bonato, M., Vernillo, G., La Torre, A., & Piacentini, M. F. (2016). Are the World Junior Championship Finalists for Middle- and Long-Distance Events Currently Competing at International Level? *International Journal of Sports Physiology and Performance, 12(3),* 316-321. <https://doi.org/10.1123/ijspp.2015-0717>

Schumacher, Y. O., Mroz, R., Müller, P., Schmid, A., & Rücker, G. (2006). Success in elite cycling: A prospective and retrospective analysis of race results. *Journal of Sports Sciences, 24(11),* 1149-1156. <http://dx.doi.org/10.1080/02640410500457299>

Sigmund, P., & Güllich, A. (2021). Individualisation, readjustment and athlete codetermination of high-performance training in athletics and volleyball (unpublished data from the study). *International Journal of Sport Science & Coaching,* advance online publication. [https://doi.org/10.1177/17479541211043183](https://doi.org/10.1177%2F17479541211043183)

Te Wierike, S. C. M. (2016). The pathway towards the elite level in Dutch basketball: A multidimensional and longitudinal study on the development of talented youth basketball players. Thesis (PhD), University of Groningen. <https://research.rug.nl/en/publications/the-pathway-towards-the-elite-level-in-dutch-basketball-a-multidi>

Tjelta, L. I., & Tjensvoll, O. (2020). All-time best Norwegian track and field athletes: to what extent did they achieve outstanding results at the ages of 15 and 18 years? *International Journal of Environmental Research and Public Health, 17(19),* 7142. <https://doi.org/10.3390/ijerph17197142>

Vaeyens, R., Güllich, A., Warr, C. R., & Philippaerts, R. (2009). Talent identification and promotion programmes of Olympic athletes (unpublished data from the study). *Journal of Sports Sciences, 27(13),* 1367-1380. <https://doi.org/10.1080/02640410903110974>

Velentza, E. (2017). A retrospective analysis of talent selection and progression within England’s rugby football union elite player performance pathway. Thesis (PhD), University of Chester. http://hdl.handle.net/10034/620558

Ward, P., Hodges, N. J., Starkes, J. L., & Williams, M. A. (2007). The road to excellence: deliberate practice and the development of expertise. *High Ability Studies 18(2),* 119-153. <https://doi.org/10.1080/13598130701709715>

Yustres, I., Martin, R., Fernández, L., & González-Ravé, J. M. (2017). Swimming championship finalist positions on success in international swimming competitions. *PLoS ONE, 12(11),* e0187462. <https://doi.org/10.1371/journal.pone.0187462>

Yustres, I., Santos del Cerro, J., Martín, R., González-Mohíno, F., Logan, O., & González-Ravé, J. M. (2019). Influence of early specialization in world-ranked swimmers and general patterns to success. *PLoS ONE, 14(6),* e0218601. <https://doi.org/10.1371/journal.pone.0218601>

Yustres, I., Santos del Cerro, J., González-Mohíno, F., Peyebrune, M., & González-Ravé, J. M. (2020). Analysis of world championship swimmers using a performance progression model. *Frontiers in Psychology, 10,* 3078. <https://doi.org/10.3389/fpsyg.2019.03078>

Zsombor, Z., Ágoston, N., & Tamás, S. (2020). Competition experience, relative age effect and average age of the senior world events’ medal-winning basketball players. *Educatio Artis Gymnasticae, 65(3),* 5-18. <https://doi.org/10.24193/subbeag.65(3)>
